# Relations Between the Printability Descriptors of Mortar and NMR Relaxometry Data

**DOI:** 10.3390/ma18133070

**Published:** 2025-06-27

**Authors:** Mihai M. Rusu, Ioan Ardelean

**Affiliations:** 1 Department of Physics and Chemistry, Technical University of Cluj-Napoca, 400114 Cluj-Napoca, Romania; mihai.rusu@phys.utcluj.ro; 2 EUT+ Institute of Nanomaterials & Nanotechnologies EUTINN, European University of Technology, European Union

**Keywords:** concrete printing, extrusion, open time, structural buildup, test methods, NMR relaxometry, accelerator, superplasticizer

## Abstract

Concrete printing technologies play a key role in the modernization of construction practices. One factor that mitigates their progress is the development of standards and characterization tools for concrete during printing. The aim of this work is to point out correlations between some printability descriptors of mortars and the data obtained from low-field nuclear magnetic resonance (NMR) relaxometry techniques. In this context, the superposed effects of an acrylic-based superplasticizer and calcium nitrate accelerator were investigated. The mortars under study are based on white Portland cement, fine aggregates, and silica fume at fixed ratios. Extrusion tests and visual inspection of the filaments evaluate the extrudability and the printing window. The selected compositions were also investigated via transverse $\left(T_{2}\right)$ and longitudinal $\left(T_{1}\right)$ NMR relaxation times. The results indicate that both additives increase the printing window of the mortar, while the accelerator induces a faster increase in specific surface area of capillary pores $\left(S/V\right)$ only after 30–60 min of hydration. Some correlations were found between the printing window and the range where the transverse relaxation rates $\left(1/T_{2}\right)$ and the pore surface-to-volume ratios $\left(S/V\right)$ increase linearly. This suggests some promising connections between NMR techniques and the study of structural buildup of cementitious materials.

## 1. Introduction

The advancement of digital fabrication with concrete (DFC) is playing a significant role in reshaping the future of civil engineering [1]. One of the most popular DFC technologies refers to extrusion-based concrete 3D printing (EC3DP). In its most developed stages, it is intended to fulfill the “fast, environmentally friendly, and economic” requirements while also enabling more complex and functional architectures and lower occupational risks [2]. This is based on the reduction in material usage and waste production, mainly due to the lack of supplementary formworks. However, challenges stem from the complex requirements of the concrete paste, the need for higher levels of quality control during the entire printing process, and the current lack of robust monitorization techniques, standards, and regulations [1,3].

Multiple studies and reviews now offer a detailed view on the printability performance of cementitious materials [4], their underlying physics [5], and the influence of different admixtures and paste formulations [6,7]. Printability performance is described using four main descriptors: pumpability, extrudability, buildability, and durability. As an overview, EC3DP relies on printable compositions that must be compatible with several processes. After the incipient mixing of the concrete ingredients, the fresh paste should be easily pumped toward an extruding nozzle. A robotic arm or gantry assures the layer-wise deposition of the material to replicate a given digital model. During this entire process, the rheological behavior of fresh cementitious materials plays a pivotal role in ensuring printability. These materials are known to behave as yield stress fluids, as they remain solid-like below a critical threshold called yield stress, while flow is initiated when this threshold is exceeded. Additionally, they exhibit thixotropic behavior: the yield stress and plastic viscosity evolve over time due to structural buildup based on reversible flocculation–deflocculation mechanisms and irreversible hydration reactions [8]. This rheological framework governs the transition of the cementitious material from a pumpable fluid to a shape-stable, buildable layer. Precise control of these transformations—especially structuration rate and drying effects—is critical also to correctly plan the printing time of each layer so to ensure structural buildability, strong interlayer bonding, and, finally, an enhanced durability of the printed element [9].

The currently available characterization techniques used in assessing the printability of concrete formulations are highlighted in multiple studies and reviews together with their drawbacks [5,6]. The studies highlight the importance of implementing other complementary techniques to EC3DP contexts. Ideally, such techniques should be robust and/or sensitive to the evolving properties of concrete during most of the printing stages, should enable non-destructive or non-contact, in situ, or in-line measurements, and should be continuous, repeatable, accurate, reproducible, scalable, user-friendly, and cost-effective [5,8]. One should also consider that investigating printable concrete using microstructure-sensitive techniques is a topic of growing interest, aiming to deepen the understanding of the relations between paste rheology, hydration dynamics, and structural development over time [9].

In this context, we investigate how proton low-field nuclear magnetic resonance (^1^H NMR) relaxometry techniques can be adapted for the characterization of EC3DP materials. NMR relaxometry is a non-contact, non-destructive technique frequently used in the analysis of a diverse palette of ^1^H-rich systems such as polymers and ionic liquids, as well as porous media like cement and other building materials [10]. It is highly adaptable as new portable, or benchtop, devices can be dispatched for monitoring small-scale or large-scale samples as well as for spatially resolved studies [10]. In the case of cementitious systems, a key advantage of applying ^1^H NMR techniques is their ability to investigate cementitious samples in both fresh [11,12,13] and hardened states [14,15]. Such measurements rely on the magnetization response of hydrogen nuclei found inside the cement pores, which greatly depends on the pore surface properties as well as the pore network features (i.e., size, connectivity, and specific surface area). During early stages of hydration, relaxometry studies can be performed to access information on the hydration dynamics, the microstructure, and pore network evolution [16,17,18]. At later stages, such techniques are usually used to investigate the porosity of the hardened samples, transport phenomena, and complex molecular dynamics at pore wall interfaces [11,19,20]. Due to these advantages, one should consider the great potential of NMR techniques in the present context of digital fabrication with concrete.

In order to prove the potential of NMR techniques for EC3DP, we initiated a set of studies intended to find correlations between NMR data and the printability of different paste compositions. Our previous investigations concerned mainly cement pastes with different admixtures and additives such as accelerators [13,20,21], pozzolanic admixtures [22,23], and superplasticizers [24]. During the present work, we will focus on some printable mortars with compositions adapted from previous studies. We selected white Portland cement (CEM I 52.5R) as a model binder due to shorter setting time and smaller concentrations of iron impurities. This is beneficial for NMR measurements since longer transverse relaxation times are obtained and superposition between different mobile water fractions is avoided. Quartz sand was introduced as a fine aggregate to obtain mortar compositions. The addition of silica fume (SF) was considered due to its known rheological and pozzolanic effects [25]. Since superplasticizers (SR) are now regularly used in EC3DP compositions [7], a commercially available SR was also introduced to enhance the workability of the fresh mix and the strength developing at later stages. However, SR also induces lower structural buildup rates and further delays the setting of cement [7]. Due to this, calcium nitrate (CN) was introduced as a set accelerator agent. The current focus is on characterizing the printability of such complex mortars using low-field ^1^H NMR relaxometry techniques. The two techniques used here are Carr–Purcell–Meiboom–Gill (CPMG) [26] for transverse relaxation measurements and Fast Field Cycling (FFC) NMR relaxometry for longitudinal relaxation measurements [27]. For the sake of simplicity, we will analyze only the effects of the superplasticizer and the accelerator on the extrudability and the hydration stages. Other optimizations concerning the mechanical performance and changes in the mixture composition, such as type of cement, inclusion of coarse aggregates and fibers, content of water, pozzolanic admixture, and aggregate size, will remain subjects for the following studies.

## 2. Materials and Methods

### 2.1. Reagents

The cement pastes were prepared using white Portland cement (CEM I 52.5 R; Holcim, Bucharest, Romania), tap water (stored at 22 °C), calcium nitrate (Ca(NO_3_)_2_ tetrahydrate; NORDIC Chemicals SRL, Cluj-Napoca, Romania), silica fume (MAPEI; Milan, Italy), and modified acrylic-based based superplasticizer Dynamon SR41 (MAPEI; Milan, Italy). The aggregate used for mortar composition consisted only of fine quartz sand with a maximum granule size of 1 mm.

The compositions that have been selected for the extrusion tests are presented in Table 1. A water-to-cement ratio, w/c = 0.3, required to achieve the standard consistency was considered as a starting point for the preparation of mortars. The SF powder substituted 5% of the cement mass and was premixed with fine aggregates. In the initial tests, fine aggregates were added to achieve aggregate-to-binder mass ratios (a/b) of 0.85 and 0.75. By considering the results of earlier studies [13,15,20], the CN content was adjusted between 0–3 wt% (by binder mass), while the SR content varied within the range of 0–1 wt% (by binder mass). The additives were premixed with water before the paste preparation, and the final solutions were poured over cement powders in a plastic vessel. A homogeneous paste was obtained by stirring continuously for 3–4 min, using a mixer.

### 2.2. Extrusion Tests

The extrudability of the cementitious pastes was evaluated using a caulk-gun setup [5]. The mortars were extruded through a 14 mm diameter nozzle. The pastes were continuously mixed at 370 rot/min for times between 15 and 120 min. The volume required to extrude a ~30 cm long filament is introduced into the extrusion chamber at different time intervals within this range. After extrusion, the filament width is measured from different regions, while the printing quality is evaluated by visual inspection in terms of crack formation, filament continuity, and texture. The extrusion tests are halted when the paste becomes too difficult to extrude or when plug formation obstructs the process. The printing window is approximated as the time during which it is possible to print filaments having an average diameter close to the nozzle diameter (14–15 mm) and standard deviations within 10%, a good adhesion to the substrate, and no apparent cracks or discontinuities. This definition is consistent with other studies where the printability window was defined as a time range when the material is extrudable and buildable, when the yield stress is within an optimal range (for given printing parameters) [28]. Below it, the shape of the paste is structurally unstable, while above it, the paste extrusion becomes difficult [28].

### 2.3. Transverse Relaxation Studies at a Given Frequency

Transverse relaxation measurements were performed using a Bruker Minispec MQ20 instrument (Bruker BioSpin GmbH; Rheinstetten, Germany) operating at 20 MHz proton resonance frequency. The samples of fresh mortar under study were poured directly into NMR tubes during the first 10 min of hydration. Relaxation time ($T_{2}$) distributions of the molecules confined inside the porous media were determined using the Carr–Purcell–Meiboom–Gill (CPMG) technique [26]. The CPMG measurements were performed periodically during the first 10 h of hydration, with a time delay of 5 min. To increase the signal-to-noise ratio, 128 echo trains were averaged for each measurement. The echo trains was comprised of 1000 echoes, recorded with short echo time intervals of 80 µs and a recycle delay of 0.5 s. The selected parameters reduce diffusion in internal gradients effect on echo train attenuation.

In the fast diffusion approximation, the effective transverse relaxation time $\left(T_{2}\right)$ of water molecules confined in pore classes of a given size and geometry depends on their surface-to-volume ratio $\left(S/V\right)$ according to the following equation:

(1) $$\frac{1}{T_{2}}=\frac{1}{T_{2}^{bulk}}+\rho \frac{S}{V}$$

where $T_{2}^{bulk}$ represents the relaxation time of unconstrained water molecules and ρ is the surface relaxivity that depends on the nature of confined molecules, the surface properties, and the measuring frequency of the NMR instrument. For heterogeneous porous systems such as mortars, the water molecules are confined in a porous network with pore sizes spanning from nanometers to hundreds of micrometers. Thus, the echo decay curves are multiexponential. To extract the transverse relaxation time distributions of the samples, inverse Laplace transforms were applied to each of the measured curves [29]. The resulting transverse relaxation time distributions can be ascribed, based on Equation (1), with pore size distributions consistent with previous works [12,30].

### 2.4. Longitudinal Relaxation Studies at Different Frequencies

As indicated above, transverse nuclear relaxation measurements performed with the CPMG technique can provide information about the surface-to-volume ratio of pores and surface relaxivity, which indirectly contains information about the interaction of molecules with the surface. However, the two parameters, relaxivity and surface-to-volume ratio, cannot be directly separated, appearing as a product in the relaxation rate described by Equation (1). This can lead to erroneous interpretations of the dependence of the relaxation rate on hydration time. Usually, this dependence is automatically attributed to the variation of the surface-to-volume ratio, when the variation of relaxivity due to modifications in the nature of the pore surface can also have the same effect. In addition, measurements performed at a single frequency cannot provide information about the characteristic times of molecular dynamics. Therefore, it is suitable that the relaxation rate be measured at different frequencies.

A technique that enables the measurement of longitudinal relaxation rates $\left(1/T_{1}\right)$ across a range of frequencies is the Fast Field Cycling (FFC) NMR relaxometry [27]. A key advantage of this technique lies in its ability to separate polarization and detection, performed at high magnetic fields for enhanced sensitivity, from the relaxation process, which occurs at lower fields. A comprehensive description of FFC NMR technique can be found in Ref. [27].

The FFC technique comprises three distinct phases: polarization, relaxation, and detection. In the first phase, the sample is polarized at high fields for a duration longer than $5T_{1}$. Then the magnetic field is rapidly changed, during a 2 ms switching time, to the relaxation field where nuclear spins are allowed to relax for variable time intervals. Following this evolution interval, the field is quickly ramped up, again to a detection field. A forthcoming 90-degree radiofrequency pulse measures the residual magnetization and thus allows determination of the relaxation time associated with the relaxation field. Because the switching times between the polarization-relaxation and relaxation-detection fields are in the millisecond range, components with significantly shorter relaxation times will not be detectable in the FFC experiment. While this is typically a drawback, we will leverage it as an advantage to selectively filter out signals corresponding to the intra- and inter-CSH phases within our cement samples and only detect the capillary component. In the present work, longitudinal relaxation rates at different frequencies were obtained via a commercial Fast Field Cycling NMR relaxometer (SMARtracer, Stelar S.R.L.; Mede, Italy), with proton frequency sweeps spanning 10 kHz to 10 MHz. All experiments were performed at a controlled temperature of 35 °C.

**Figure 1:**
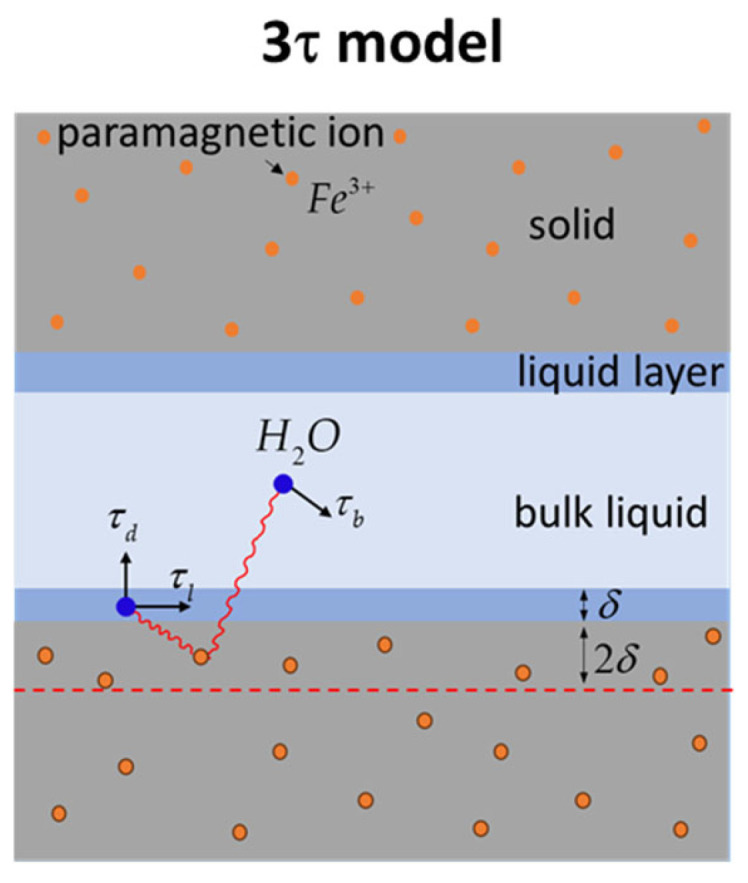
Schematic illustration of the 3-Tau model [27,31,32,33].

While CPMG relaxation measurements, accomplished at a fixed-proton resonance frequency and analyzed via a straightforward equation relating surface-to-volume ratio to relaxation time, are frequency-independent, FFC NMR experiments demand a different analytical strategy. The frequency-dependent longitudinal relaxation rates acquired through FFC, known as relaxation dispersion curves, necessitate the use of a tailored relaxation model for accurate dynamic parameter extraction. For cement-based materials, the 3-Tau model, developed and tested by Faux and collaborators [31,32,33], provides a robust framework for interpreting water molecule relaxation within confined pores. This model, schematically depicted in Figure 1 and extensively explored in the publications of Faux et al. [20,31,32,33,34,35,36], will be outlined here, focusing on its key aspects. Additionally, a fitting tool, created and tested by Faux et al. [37], is available to facilitate the implementation of the 3-Tau model on experimental data.

According to the 3-Tau model, the relaxation rate of water protons confined inside porous materials is primarily determined by the thermally modulated dipolar interactions between proton spins and the electronic spins of the paramagnetic Fe^3+^ ions of the solid matrix. As shown in Figure 1, water molecules occupy two distinct environments inside pores: a bulk-like state and a surface monolayer of a thickness δ=0.27 nm. The random displacement of water molecules within these environments, both along surfaces and in the bulk (represented by arrows in Figure 1), modulates the dipolar interaction, leading to a longitudinal relaxation rate that depends on three characteristic time constants (3-Tau), the concentration of paramagnetic ions, and the surface-to-volume ratio:

(2) $$\frac{1}{T_{1}\left(\omega \right)}=f\left(\tau_{l},\tau_{b},\tau_{d},N_{para},\frac{S\delta }{V}\right)$$

Within the aforementioned model, $\tau_{l}$ represents a characteristic time associated with water molecule movement along the surface, and it is linked to the surface water diffusion coefficient $D_{l}$ through the formula $\tau_{l}=\delta^{2}/6D_{l}$. Similarly, $\tau_{b}$ defines the diffusion time constant for bulk-like water molecules, describing their displacement over a distance δ, and is related to the bulk diffusion coefficient $D_{b}$ via $\tau_{b}=\delta^{2}/6D_{b}$. It is worth noting that pure water at ambient temperature exhibits a $\tau_{b}$ of 5.3 ps, but this value is expected to increase within cement paste pores. This increase arises from the presence of dissolved ions, which impedes water diffusion, and the reduced mobility of water molecules within the secondary hydration layer at the surface, which significantly influences bulk relaxation [36]. Assuming a desorption process, described by an exponential formula $\exp\left(-t/\tau_{d}\right)$, $\tau_{d}$ quantifies the residence time of water molecules at the surface before desorption. 

In this analysis, the concentration of paramagnetic ions, denoted as $N_{para}$, is treated as an effective parameter, describing the average numerical density at a depth 2δ beneath the pore surface, as indicated by the dashed line in Figure 1. It is important to observe that if hydration products accumulate on the cement grain surfaces, $N_{para}$ becomes only indirectly related to the original paramagnetic impurity levels within the cement-based material. The ratio δS/V between the volume of the surface layer and the pore volume, extracted from the fitting approach, can be utilized to estimate pore dimensions, assuming a planar pore geometry. This ratio facilitates the determination of pore size as the spacing between parallel planes. However, for materials like hydrating cement, which exhibit dynamic pore structures, changes in S/V can also reflect variations in fractal dimension [13] and further hypothesize their direct correlation with the process of structural buildup characteristic of cementitious pastes. 

## 3. Results and Discussions

### 3.1. Extrudability Tests

**Figure 2:**
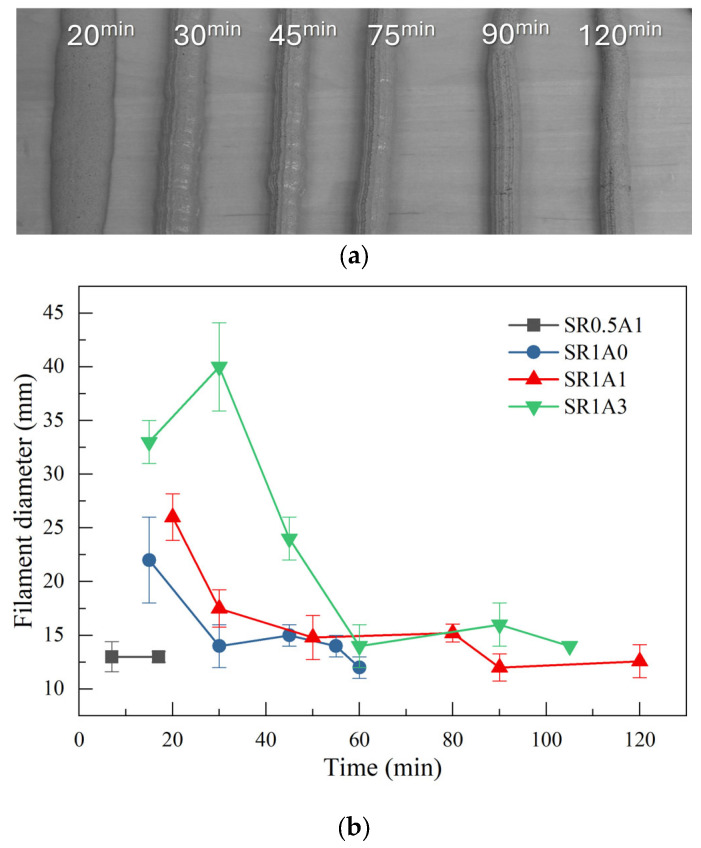
(**a**) Filaments extruded at different hydration times using the SR1A1 mortar. (**b**) Extrusion test results obtained for the investigated mortars.

Extrusion tests can offer a first insight into the printability of the investigated mortars. An example of extruded filaments is shown in Figure 2a, which is characteristic of the SR1A1 mortar. During extrusion, at short hydration times, the two asymptotic regimes that describe mortar extrusion could be observed. Initially, the free flow regime can be observed, which is characteristic of more fluid compositions. In this case, the filament diameter is strongly affected by flow or gravity. At later hydration times, the mortar transits into the infinite brick regime, typically associated with stiffer compositions, where the filament cross-section is predominantly defined by the nozzle geometry [3].

The changes induced by the presence of SR and CN additives greatly influence the transition from one regime to another, as reflected in the printability window of each paste composition. The extrusion test results are summarized in Figure 2b and Table 2. The mortars with lower SR or higher aggregate content had a poor extrudability since nozzle blockage was obtained a few minutes after mixing. One can observe that the presence of CN also drastically changes the printability window. At higher CN dosage, it can be observed that the free flow regime is prolonged as more flowable mixtures are obtained. During this regime, the nozzle-induced shape of the filament is lost since the low-yield stress of the mortars is exceeded by gravitational forces [3]. In terms of filament quality, start, and duration of the printing window, the best performance was obtained for the SR1A1 mortar. The free flow regime ended at 35 ± 5 min, and the duration of the printing window was estimated as 40 ± 10 min. At a CN dosage of 3%, the onset of the printing window is delayed until ~60 min, and longer printing windows can be obtained. One should note that the printing windows depends also on the extrusion installation. This is why we estimated the onset and offset times considering a tolerance within 5 and 15 min, respectively.

### 3.2. Probing Printable Mortars via NMR-CPMG

#### 3.2.1. The Transverse Relaxation Time Distribution During Cement Hydration

**Figure 3:**
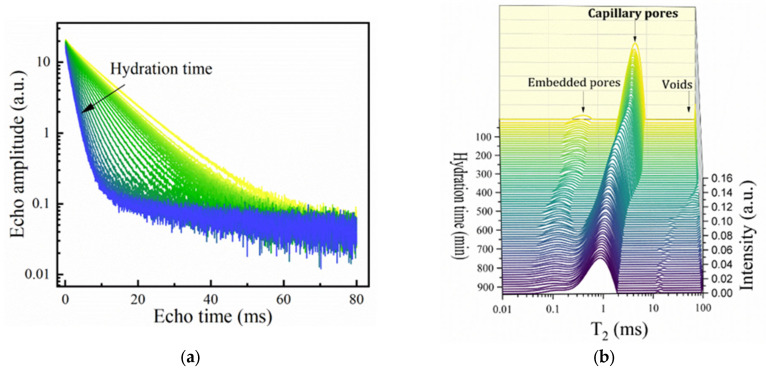

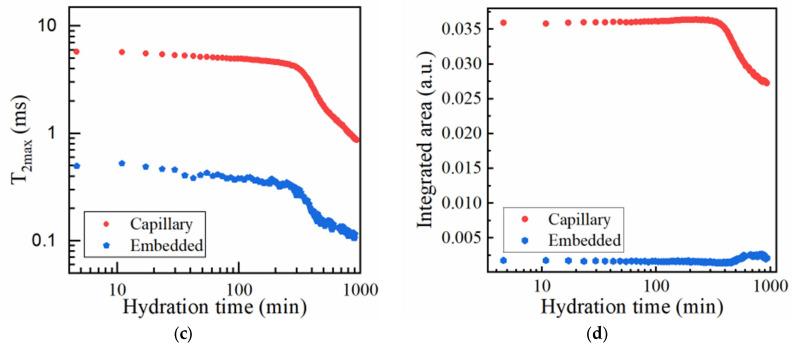
Monitorization of mortar hydration using CPMG: (**a**) Echo time decay curves obtained at different hydration times for sample SR1A0. The arrow is oriented from lower to longer hydration times. (**b**) Corresponding Laplace spectra where the $T_{2}$ contributions are attributed to water from capillary pores (main contribution), embedded water, and water from voids. (**c**) Evolution of the “peak centers.” (**d**) Integrated intensities for the capillary and embedded water contributions.

To evaluate the open time from the perspective of the microstructural evolution during the hydration, the samples that passed the extrusion tests (an open time larger than 20 min) were further investigated using advanced NMR techniques. One should note that in the present context, the cement chemistry abbreviations are used, where C = CaO, S = SiO_2_, A = Al_2_O_3_, F = Fe_2_O_3_, and H = H_2_O [13]. Notations such as C_3_S, C_3_A and CSH, and NO_3_-AFm will refer to tricalcium silicate (alite), tricalcium aluminate, calcium silicate hydrate, and calcium monosulfoaluminate. An example of the multiexponential CPMG echo train decay curves and the associated $T_{2}$ relaxation time distributions is represented for sample SR1A0 at different hydration times in Figure 3a,b, respectively. One should consider that, given the maximum echo time of 80 µs that can be achieved, only the mobile water contributions with longer relaxation times were detectable.

During the first hydration stages of Portland cement, the main contribution to the Laplace spectrum is observed in the range between $T_{2}∼10^{0}-10^{1}\;\mathrm{ms}$ and is strongly influenced by w/c ratios. This is initially associated with water molecules confined within capillary pores [38]. The second contribution $\left(T_{2}∼10^{-1}-10^{0}\;\mathrm{ms}\right)$ is initially associated with water embedded in smaller pores that form, i.e., inside the cement grain agglomerates [38] or within the network of the primary hydrate phases. We will refer to this mode as originating from “embedded water.” The smallest relaxation times that can be measured by CPMG $\left(T_{2}∼10^{-1}\;\mathrm{ms}\right)$ are associated with mobile water diffusing within the lamellar CSH. This pore class is frequently addressed in cement literature as “intra-CSH pores” [30]. Other $T_{2}$ modes originating from water inside voids or macroscopic pores may be detected at $10^{1}-10^{2}\;\mathrm{ms}$ but can be neglected due to their small contribution.

The evolution of the “peak centers,” denoted $T_{2\max}$, and integrated intensities are presented in Figure 3c,d for the main capillary and the hydrated gel modes, as measured for sample SR1A0. One can observe that, in the initial hydration stages, the signals specific to the gel pores are less intense and show a similar behavior as the capillary peak. This will take place mainly until the end of the dormancy stage.

Since the dormant period is most relevant to the present context, our analysis is limited to the data associated with the main capillary peak. In the study of different cement and mortars, two different time intervals can be observed during which $T_{2\max}$ decreases. The first interval, also called initial stage, spans roughly between 5 and 80 min after hydration is initiated. Hypothetically, this can be influenced by the complex set of reactions taking place between mixing and induction stages, in terms of water loss, flocculation, and early hydrate nucleation [39]. During this stage, the primary phases such as metastable CSH [40] and the gypsum-mediated ettringite needles [41,42] are developing from the hydration of C_3_S (slow kinetics) and C_3_A (fast dissolution), respectively. This plays a significant role in the structural buildup of the cementitious system. The next time interval is specific to the dormant stage when the C_3_S reactivity is inhibited. A steady-state decrease in the $T_{2\max}$ can be observed. During dormancy, the integrated intensity can exhibit a plateau, a steady decrease, or, in some cases, even a slow Gaussian-like increase for different compositions, mostly influenced by the changes in the distribution of water populations. With the progress of hydration into the acceleration period, the capillary mode shifts to lower values and will be more characteristic of mobile water from inter-hydrate spaces [25]. The accelerated CSH growth is initiated, and the setting of cement takes place. The NMR setting time is defined here as the inflexion point of the $T_{2\max}$ curve versus time, as suggested in other studies [43].

#### 3.2.2. Effects Introduced by SR and CN over the Hydration Kinetics

**Figure 4:**
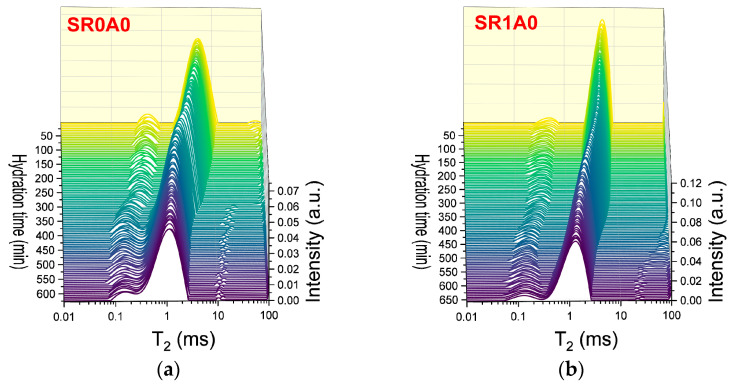

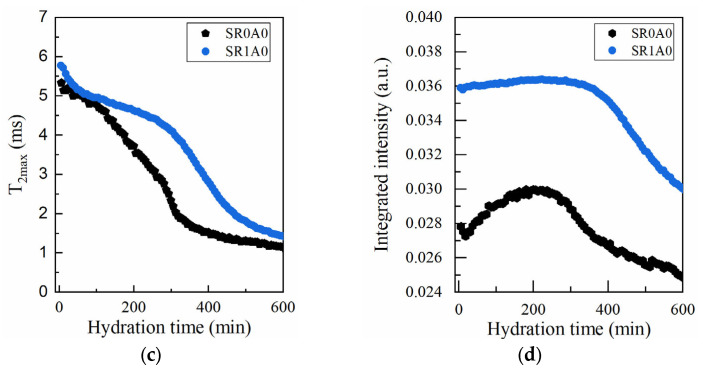
CPMG investigations on the effects induced by SR: Laplace spectra of (**a**) SR0A0, (**b**) SR1A1 mortars, (**c**) evolution of the $T_{2\max}$, and (**d**) integrated intensities of the capillary mode for both samples.

The effects of SR addition, as revealed by CPMG measurements, are presented in Figure 4. One of the primary effects observed is an increase in the initial probability densities (reflected by the peak height and integrated area) and in the characteristic $T_{2\max}$ of the capillary mode. The second effect is the slower hydration during induction and dormancy stage. The steady decrease in $T_{2\max}$ ends below 100 min for SR0A0, while for SR1A0, this trend continues until approximately 250 min. The presence of SR will trigger a delay in the setting time. Sample SR0A0 reveals an inflexion point around 150 min, while the setting of SR1A0 is observed around 365 min. Also, the maximum in integrated area observed in Figure 4d is broader, and it is also delayed when SR is introduced, which is presumably attributed to lower water consumption rates. All of these observations are corroborated with the known effects of the superplasticizer: improved dispersion of particles, improved fluidity and workability, and retarded hydration. As proposed by Jansen et al., the governing physical mechanisms involve the polymer adsorption onto grain surfaces or hydrate nucleation sites and the SR complexation of Ca^2+^ ions within the pore solution [44].

**Figure 5:**
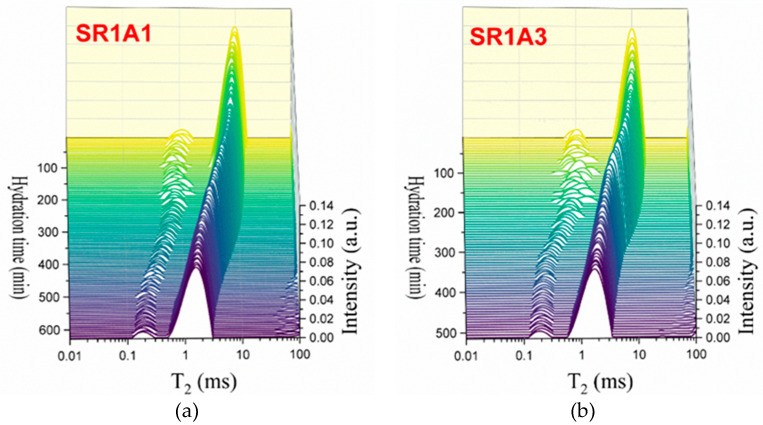

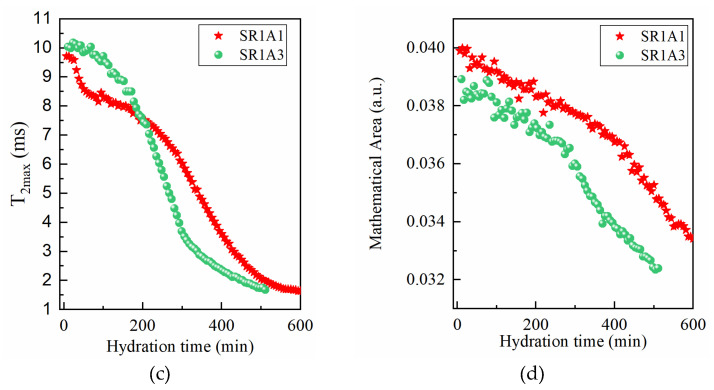
CPMG investigations on the effects induced by CN: Laplace spectra of (**a**) SR1A1, (**b**) SR1A3 mortars, (**c**) evolution of the $T_{2\max}$, and (**d**) integrated intensities of the capillary mode for both samples.

As observed in Figure 5, the presence of CN influences the hydration process during each of the early stages. The Laplace spectra indicated in Figure 5a,b reveal more fluctuations in the hydrated gel mode at early stages, as compared to SR0A0 and SR1A1. From the first minutes of hydration, the transverse relaxation time associated with capillary pores (Figure 5c) is shown to increase with CN content. This was observed in previous studies performed on plain cement samples as well [20]. Also, one should note that, as opposed to the trend observed for SR0A0, the presence of CN counteracts the initial decrease in $T_{2\max}$, usually observed within the first 100 min. Indeed, the main mechanisms involved in this stage are assigned with changes in the initial ionic solution composition and increased zeta potentials [45], sulfate precipitation, and development of ettringite and NO_3_-AFm phases [46]. The increase in the workability of cement pastes and the variations in needle morphologies were also confirmed in previous studies on CN-modified cement pastes as well [15].

The role of the CN as a set accelerator is revealed near the end of the dormancy stages: when both $T_{2\max}$ and integrated areas decrease at higher rates in the presence of CN. The inflexion point of the $T_{2\max}$ curve, associated with the initial setting of cement, is also shifted toward lower values. These indicate faster water consumption and growth of hydrates.

### 3.3. Probing the Effect of the Accelerator via NMR-FFC

To better understand the role of the accelerator, Fast Field Cycling NMR measurements were performed on SR1A1 and SR1A3 mortars and further attempted to compare the results with the data obtained during extrusion tests and CPMG measurements.

To examine the relaxation behavior over time, both samples underwent longitudinal relaxation analysis utilizing the established FFC methodology. A switching time of 2 ms between polarization-relaxation and relaxation-detection was employed to minimize the influence of intra- and inter-CSH water and thus detect only the signal from capillary water. As can be seen from the transverse relaxation time distributions shown in Figure 4a,b and Figure 5a,b, for evolution times significantly longer than the transverse relaxation times corresponding to the small peaks in these figures, the contribution of these components to the signal can be neglected. It is also worth noting that our previous studies on samples subjected to a desaturation process—where only the water in the intra- and inter-CSH pores remained—did not allow the detection of an NMR signal using the FFC technique.

**Figure 6:**
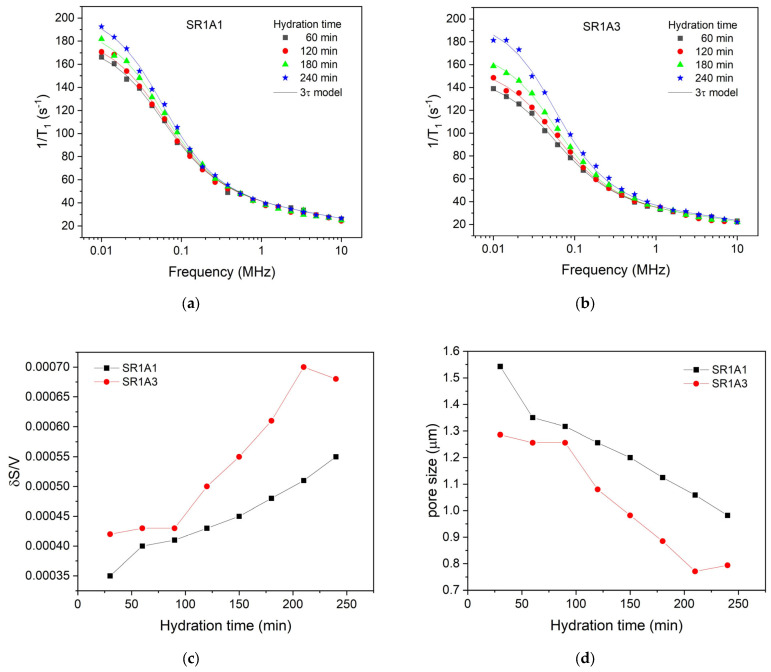
Selected relaxation dispersion curves for the two samples, SR1A1 (**a**) and SR1A2 (**b**), containing different amounts of accelerator. The continuous lines represent the best fit with the 3-Tau model. Parameters extracted from the fitting approach allowed monitoring of the surface-to-volume ratio (**c**) and the pore size (**d**) during hydration.

The initial relaxation dispersion profile was acquired 30 min post-mixing, accounting for necessary instrument stabilization. Subsequent profiles were generated every 30 min, ending in a final measurement at 240 min. Figure 6 shows selected relaxation dispersion curves recorded for the two samples SR1A1 (Figure 6a) and SR1A3 (Figure 3b), respectively. The best-fitting curves with the 3-Tau model of Faux and collaborators [31,32,33,34,35] are also indicated as continuous lines. The fitting procedure employed in this study was based on the methodology outlined in Ref. [36], with computations performed using the software developed by Kogon and Faux [37].

For fitting the experimental data, we proceeded as follows: initially, all parameters were set free. The fitting curves overlapped remarkably with the experimental data, but we found that the results were inconclusive and observed a certain repetition of the same parameter for different hydration times. Then, after several rounds of fitting and maintaining certain parameters constant, we noted that the data could be fitted well with the same set of parameters. Thus, it was found that all dispersion curves can be fitted by maintaining the parameters $\tau_{l}=0.31\;\mu s$ and $\tau_{D}=5.62\;\mu s$ for both samples and all hydration times. This shows that the affinity of the pore surface for water molecules; therefore, the nature of the surface did not change during the dormancy stage and remains the same for both samples. Note, however, the parameter $\tau_{b}$, which is associated with the mobility of water molecules inside the capillary pores, could not be used as the same for samples SR1A1 and SR1A3. Thus, $\tau_{b}=15.4\;\mathrm{ps}$ was extracted for sample SR1A1, throughout the studied hydration interval, and $\tau_{b}=17.8\;\mathrm{ps}$ for sample SR1A3 in the interval 30–210 min. This indicates a lower mobility of water molecules in sample SR1A3 due to a higher amount of accelerator present in the sample. In addition, at a hydration time of 240 min, in the case of sample SR1A3, a decrease in the parameter $\tau_{b}$ to a value of 15.4 ps was observed, which can be associated with a decrease in the concentration of calcium ions in the solution.

Another parameter extracted from the fitting procedure is $N_{par}$, which has a value of $0.008{\;\mathrm{ions}/\mathrm{nm}}^{3}$ for sample SR1A1, throughout the hydration interval between 30 min and 240 min. In the case of sample SR1A3, the parameter $N_{par}$ has a value of $0.006{\;\mathrm{ions}/\mathrm{nm}}^{3}$ for the hydration interval of 30–210 min. Here too, a change in this parameter to a value of $0.007{\;\mathrm{ions}/\mathrm{nm}}^{3}$ was obtained for the hydration time of 240 min. The generally lower value of the parameter $N_{par}$ for sample SR1A3 compared to SR1A1 indicates more substantial precipitation of hydration products, such as ettringite, on the pore surface, which isolates the paramagnetic ions and thus offers an apparently lower $N_{par}$. However, in the case of sample SR1A3 at the hydration time of 240 min, an increase in this parameter is observed, which can be explained by a better accessibility of the molecules to the pore surface and the penetration of the initially uniformly deposited ettringite layer.

A further outcome of the fitting procedure is the examination of the surface-to-volume ratio for both samples throughout the hydration process. As illustrated in Figure 6c, the surface-to-volume ratio is consistently higher for sample SR1A3, which contains a greater amount of accelerator compared to sample SR1A1. This observation suggests the presence of smaller pores, as exhibited in Figure 6d, but may also be attributed to a larger fractal dimension in the SR1A3 samples, as previously noted in Ref. [13]. Notably, we also observe a more rapid evolution of the surface-to-volume ratio in the sample with higher accelerator content, a result that aligns with the estimations derived from the CPMG technique described earlier. It is important to highlight that, in CPMG measurements, the faster evolution of the transverse relaxation rate in SR1A3 compared to SR1A1 could stem from both changes in the surface-to-volume ratio of the capillary pores and variations in relaxivity. However, FFC measurements demonstrate that the relaxivity, associated with pore surface properties, remains constant throughout the studied time interval, as the characteristic times $\tau_{l}$ and $\tau_{D}$ remain the same for both samples. Consequently, the observed variation in relaxation rate with hydration time can be attributed mainly to alterations in the surface-to-volume ratio of the pores. This result highlights a significant advantage of the FFC technique over CPMG.

### 3.4. NMR and Printability Descriptors

During the CPMG and FFC measurements, one can observe that the CPMG routine is faster and more user-friendly, and the data processing steps are less pretentious. The disadvantage is that the obtained transverse relaxation time $T_{2}$ depends both on the relaxivity ρ and the surface-to-volume ratio $\left(S/V\right)$; hence, direct information on S/V and, consequently, the effective pore size cannot be directly determined. A first attempt to lift this constraint is using FFC results to discriminate between relaxivity and specific surface area as one possible route to resolve the limitations of the CPMG technique and end this section by mentioning the limitations and future studies.

**Figure 7:**
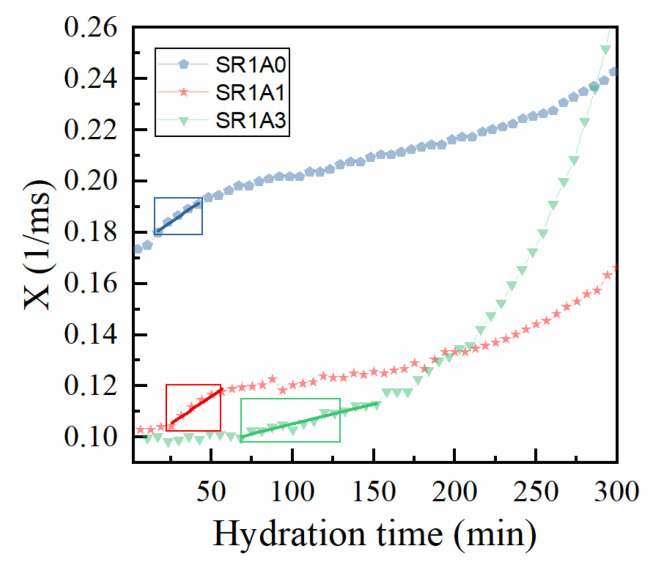
Correlation between extrusion tests and the CPMG results on the effects introduced by CN. The evolution of the relaxation rates specific to the capillary mode (*X =* $1/T_{2\max}$) are represented by bullet points. The regions of interest (rectangles) correspond with the printing windows determined from extrusion tests. The continuous lines correspond with linear fits of X versus time.

For simplicity, we further denote the relaxation rate $1/T_{2\max}$ as X and represent it as a function of hydration time, as shown in Figure 7. One can observe a complex behavior best described by three regimes: (1) where X is almost constant (associated with the free flow regime), (2) a linear increase in X (similar to a thixotropic behavior), followed by (3) a quasi-exponential increase. Indeed, by superposing the printing window obtained from extrusion tests (rectangular boxes in Figure 7), one can distinguish a correlation between this and the linear regime for the relaxation rate. If this range is fitted by a linear function (see continuous lines in Figure 7):

(3) $$X=X_{0}+\gamma t$$

One might be able to evaluate the printing window and to measure the “CPMG-derived structuration rate” played by the γ parameter. The fit results are presented in Table 3. Sample SR1A1 appears to have the highest γ value, followed closely by SR1A0. At increased accelerator dosages, the onset of the open time is delayed, as observed during the extrusion tests, and smaller γ values are determined during this regime. As shown in Figure 7, for SR1A3 compositions, the structural buildup will significantly increase only after 150 min. The results indicate that, with respect to cement–CN mixtures [13], the accelerator activity is slower when including superplasticizers. While lower structuration rates (and γ values) during printing may increase the printability window and probably improve the inter-layer adhesion [8], it might negatively affect the buildability when fast printing rates are used.

**Figure 8:**
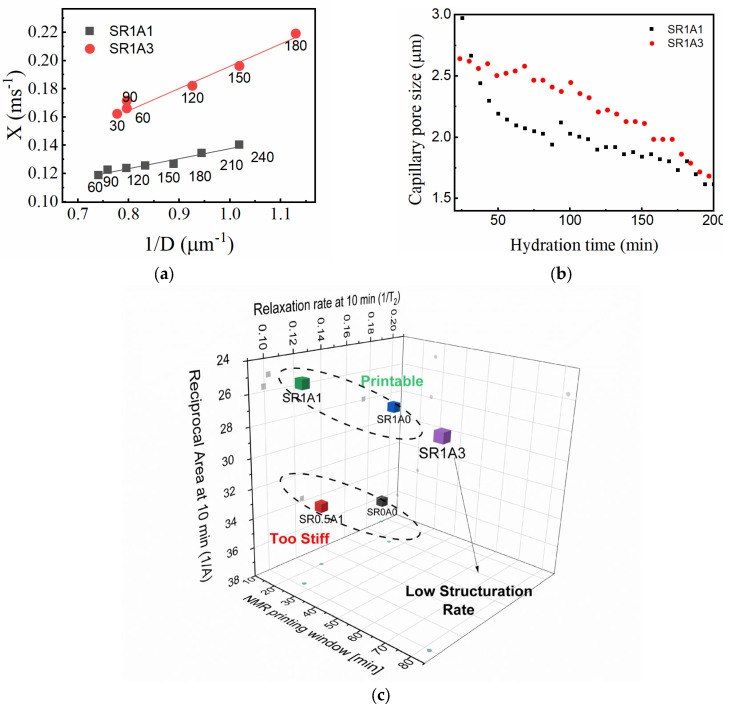
(**a**) Determination of relaxivity constants by the linear fit of the transverse relaxation rate $\left(1/T_{2}\right)$ versus the inverse of particle size 1/D obtained from FFC. (**b**) Effective particle size as determined from CPMG. (**c**) Classification of mortar compositions using the inverse of the integrated intensity $\left(1/A\right)$ and relaxation rate $\left(1/T_{2}\right)$ at 10 min and the NMR-derived printing window.

The γ parameter describes the evolution of the relaxation rate with hydration time, and as previously mentioned, it may be difficult to directly interpret it as a structuration rate due to the unknown contributions from the relaxivity term (see Equation (1)). However, under the assumptions of a certain pore geometry and using data concerning capillary pore size, one may be able to lift these constraints. To determine the relaxivity of SR1A1 and SR1A3 compositions, we will assume a slit pore geometry and propose to use the pore size determined by FFC (Figure 6d) and $T_{2\max}$ values determined from CPMG as input in Equation (1). Then, the transverse relaxation rate can be expressed against the inverse pore size (1/D), as shown in Figure 8a. Based on Equation (1), the linear fit results indicate the relativity values for SR1A1 and SR1A3 of 35 and 77 μm/s and the bulk relaxation rates $\left(1/T_{2b}\right)$ of 0.07 and 0.04 ms^−1^, respectively. The linear fit was performed only during the time interval associated with the dormancy stage (hydration time indicated as labels in Figure 8a), when a fairly constant relaxivity can be assumed. Using this approach, one can estimate the effective capillary pore size from CPMG measurements as shown in Figure 8b. The rate of evolution of the capillary pore S/V is obtained by dividing γ by ρ. During dormancy, the values obtained for SR1A1 and SR1A3 are 0.011 and 0.002 (μm∙min)^−1^, respectively. The influence of CN over the pore size evolution trends is similar for the FFC and CPMG results. However, the FFC technique indicates smaller pores in SR1A3 than in SR1A1, while the CPMG data reveals larger pores on the same hydration time interval. One should note that the capillary pore dimensions determined from CPMG are close to the values obtained from SEM measurements performed on CN-containing cements [15]. We expect that the observed differences are due to diffusion effects and that the approximations and the fitting models are perhaps oversimplified. Even though the analysis offers a great deal of details regarding the structuration process, future studies are needed for further refinement.

As an overview, the NMR response is strongly dependent both on surface properties of the particles and the pore fraction scanned by mobile water molecules. According to the available models reviewed in Ref. [47], the rheologically determined structural buildup shares a very similar spectrum of variables. This means that both ^1^H NMR and the rheologic response are influenced by the colloidal interactions and hydration reactions found in cementitious mixtures. We propose one protocol that can aid in roughly classifying mortars as printable or non-printable. One promising classification is based on the relaxation rate $\left(1/T_{2}\right)$, reciprocal area under the capillary peak $\left(1/A\right)$ measured at 10 min, as shown in Figure 8c. Since the first two parameters are dependent on S/V and the population of water-filled capillary pores, larger values of $1/T_{2}$ and 1/A are signs of increased stiffness (higher yield stresses). A third parameter, such as NMR-derived printing window or structuration rate, could reflect the buildability of the analyzed composition. The CPMG-based approach is fast and sensitive to the transformations taking place in printable mortars. A detailed analysis of the precision and repeatability of CPMG measurements is presented in the Supplementary Materials. The analysis includes also the effects induced by different factors such as temperature, spatial inhomogeneities, and mechanical transformations applied to the paste sample. Also, the method is consistent with other complimentary tests. One can observe in the Supplementary Materials a parallel between temperature readings from the core of SR1A0, SR1A1, and SR1A3 mortar samples, ultrasound pulse velocity determined during UPV tests, and the relaxation rates. Thus, we believe the current study demonstrates the potential of ^1^H NMR techniques as effective tools for characterizing and monitoring printable mortars. However, cross-correlation studies and the use of other complementary techniques will be essential in future research to further refine the NMR-based characterization of structural buildup, as well as the mortar classification protocols.

## 4. Conclusions

The present study was centered on using ^1^H NMR techniques as tools for the characterization of mortars for EC3DP applications. The main advantage is that they represent non-contact approaches able to analyze cementitious samples during all hydration stages.

Here, the effects introduced by superplasticizer and accelerator admixtures over the mortar paste extrudability were monitored using CPMG and FFC relaxometry and correlated with typical extrusion tests. A commercial acrylic-based superplasticizer and calcium nitrate were introduced as aqueous solutions to dry mixtures based on white Portland cement (CEM I 52.5 R), silica fume, and fine aggregates.

The extrusion tests were performed to identify the printable compositions. The tests revealed that adding calcium nitrate (1–3% by mass of binder) and superplasticizer will increase the paste fluidity, thus delaying and extending the printability window.

Fast measurements of spin echo decays were performed using the CPMG technique. The signal amplitude is sensitive to mobile water quantities and the transverse relaxation rate to specific surface areas of the evolving pores. The study suggests that CPMG data is also sensitive to flocculation, hydration, stiffening, and setting processes and, hence, can serve in the analysis of structural buildup of cements.

The induction, dormancy, and acceleration stages of the hydrating mortars were identified from $T_{2\max}$ versus time plots. The obtained data confirm that the prolonged dormancy and delayed setting due to the superplasticizer–accelerator mixture and that cement setting will occur faster with the increase in the accelerator dosage. The increased $T_{2}$ values and the fluctuations observed in the integrated intensity at higher CN dosage are signs of higher paste fluidity and an unstable pore network, mainly due to colloidal interactions. Also, the relaxation rate can be adopted as a good representation for structural buildup. The regime where it increases linearly is well superposed with the printing window determined from the extrusion tests. This suggests the possibility to use CPMG as an alternative route to investigate the structural buildup of cementitious materials.

The main limitation for this approach refers to the mutual dependence of $T_{2}$ on relaxivity and surface-to-volume ratio. This was counteracted by using FFC relaxometry measurements interpreted by a 3-Tau model. Essentially, it was observed that, on short intervals specific to the dormancy stage, the capillary pore surface properties, and thus the relaxivity, can be considered as constant. The effects introduced by the admixtures over the effective S/V determined from FFC show similar trends as seen from extrusion and CPMG results.

The CPMG data was also used to roughly classify the systems as printable and non-printable mixtures using the relaxation rate and the reciprocal intensity measured at 10 min. The NMR-derived printing window was used as a third classifier.

Future studies are needed to deeper understand and refine the link between ^1^H NMR data and the paste rheology and strength development of printable cementitious materials.

## Figures and Tables

**Table 1:** Composition of the investigated mortars.

Sample	w/b	a/b	SF (%)	SR (%)	CN (%)
M04	0.4	0.85	5	0	0
SR0A0	0.3	0.75	0	0	0
SR0.5A1	0.3	0.75	−5	0.5	1
SR1A0	0.3	0.75	−5	1	0
SR1A1	0.3	0.75	−5	1	1
SR1A3	0.3	0.75	−5	1	3

w/b and a/b = water/binder and fine aggregate-to-binder mass ratios, CN = accelerator, SR = superplasticizer, SF = silica fume, expressed as percentages by mass of substitution (SF) or addition (CN, SR) relative to mass of cement.

**Table 2:** Extrusion test results obtained for the entire set of investigated mortar compositions.

Sample	T_start_	T_stop_	Printing Window (min)	Extrudability
M04	10	-	n.a.	Failed
SR0A0	10	20	<10 min	
SR0.5A1	5	20	<10 min	
SR1A0	15	40	25	Passed
SR1A1	35	75	40	
SR1A3	60	120	60	

T_start_ and T_stop_ represent the onset and endset times of the printing window.

**Table 3:** Linear results for interpreting the relaxation rate evolution on the open time interval.

Sample	T_start_ (min)	T_end_ (min)	X_0_ (1/ms)	ɣ (1/ms)/min	ɣ/ρ (µm∙min)^−1^
SR1A0	20	40	0.173	4.3 × 10^−4^	-
SR1A1	25	50	0.093	4.9 × 10^−4^	0.011
SR1A3	70	150	0.089	1.6 × 10^−4^	0.002

The uncertainty for Tstart and Tend considered during fitting is ±5 min; the standard deviations of intercept X_0_ and slope ɣ were 0.001 and 0.4 × 10^−4^, respectively, while the adjusted R^2^ values were above 0.94.

## Data Availability

The original contributions presented in this study are included in the article/Supplementary Materials. Further inquiries can be directed to the corresponding author.

## Supplementary Materials

The following supporting information can be downloaded at: https://www.mdpi.com/article/10.3390/ma18133070/s1, Figure S1: Effects induced over $T_{2}^{\max}$ and integrated intensity by temperature (a,b), sampling depth (c,d), and mechanical transformations (e,f) illustrated for SR1A1 mortar; Figure S2: Paralell between the evolution of relaxation rate (a) and ultrasound pulse velocity measured during UPV tests.

## References

[1] Ma G., Buswell R., Leal da Silva W.R., Wang L., Xu J., Jones S.Z. (2022). Technology Readiness: A Global Snapshot of 3D Concrete Printing and the Frontiers for Development. Cem. Concr. Res..

[2] De Schutter G., Lesage K., Mechtcherine V., Nerella V.N., Habert G., Agusti-Juan I. (2018). Vision of 3D Printing with Concrete—Technical, Economic and Environmental Potentials. Cem. Concr. Res..

[3] Wangler T., Roussel N., Bos F.P., Salet T.A.M., Flatt R.J. (2019). Digital Concrete: A Review. Cem. Concr. Res..

[4] Buswell R.A., Leal de Silva W.R., Jones S.Z., Dirrenberger J. (2018). 3D Printing Using Concrete Extrusion: A Roadmap for Research. Cem. Concr. Res..

[5] Fasihi A., Libre N.A. (2024). From Pumping to Deposition: A Comprehensive Review of Test Methods for Characterizing Concrete Printability. Constr. Build. Mater..

[6] Rahman M., Rawat S., Yang R.C., Mahil A., Zhang Y.X. (2024). A Comprehensive Review on Fresh and Rheological Properties of 3D Printable Cementitious Composites. J. Build. Eng..

[7] Marchon D., Kawashima S., Bessaies-Bey H., Mantellato S., Ng S. (2018). Hydration and Rheology Control of Concrete for Digital Fabrication: Potential Admixtures and Cement Chemistry. Cem. Concr. Res..

[8] Reiter L., Wangler T., Roussel N., Flatt R.J. (2018). The Role of Early Age Structural Build-up in Digital Fabrication with Concrete. Cem. Concr. Res..

[9] Roussel N., Lowke D. (2022). Digital Fabrication with Cement-Based Materials.

[10] Nagel S.M., Strangfeld C., Kruschwitz S. (2021). Application of 1H Proton NMR Relaxometry to Building Materials—A Review. J. Magn. Reson. Open.

[11] Pop A., Bede A., Dudescu M.C., Popa F., Ardelean I. (2016). Monitoring the Influence of Aminosilane on Cement Hydration Via Low-Field NMR Relaxometry. Appl. Magn. Reson..

[12] Rusu M.M., Vilau C., Dudescu C., Pascuta P., Popa F., Ardelean I. (2023). Characterization of the Influence of an Accelerator upon the Porosity and Strength of Cement Paste by Nuclear Magnetic Resonance (NMR) Relaxometry. Anal. Lett..

[13] Ardelean I. (2021). The Effect of an Accelerator on Cement Paste Capillary Pores: NMR Relaxometry Investigations. Molecules.

[14] Nicula L.M., Corbu O., Ardelean I., Sandu A.V., Iliescu M., Simedru D. (2021). Freeze–Thaw Effect on Road Concrete Containing Blast Furnace Slag: NMR Relaxometry Investigations. Materials.

[15] Rusu M.M., Vulpoi A., Vilau C., Dudescu C.M., Păşcuţă P., Ardelean I. (2022). Analyzing the Effects of Calcium Nitrate over White Portland Cement: A Multi-Scale Approach. Materials.

[16] Bligh M.W., d’Eurydice M.N., Lloyd R.R., Arns C.H., Waite T.D. (2016). Investigation of Early Hydration Dynamics and Microstructural Development in Ordinary Portland Cement Using 1H NMR Relaxometry and Isothermal Calorimetry. Cem. Concr. Res..

[17] Liu H., Sun Z., Yang J., Ji Y. (2021). A Novel Method for Semi-Quantitative Analysis of Hydration Degree of Cement by 1H Low-Field NMR. Cem. Concr. Res..

[18] Kurihara R., Maruyama I. (2022). Surface Area Development of Portland Cement Paste during Hydration: Direct Comparison with 1H NMR Relaxometry and Water Vapor/Nitrogen Sorption. Cem. Concr. Res..

[19] Papp V., Janovics R., Péter Kertész T., Nemes Z., Fodor T., Bányai I., Kéri M. (2023). State and Role of Water Confined in Cement and Composites Modified with Metakaolin and Additives. J. Mol. Liq..

[20] Rusu M.M., Faux D., Ardelean I. (2023). Monitoring the Effect of Calcium Nitrate on the Induction Period of Cement Hydration via Low-Field NMR Relaxometry. Molecules.

[21] Simedru A.F., Becze A., Cadar O., Scurtu D.A., Simedru D., Ardelean I. (2023). Structural Characterization of Several Cement-Based Materials Containing Chemical Additives with Potential Application in Additive Manufacturing. Int. J. Mol. Sci..

[22] Toma I.-O., Stoian G., Rusu M.-M., Ardelean I., Cimpoeşu N., Alexa-Stratulat S.-M. (2023). Analysis of Pore Structure in Cement Pastes with Micronized Natural Zeolite. Materials.

[23] Crețu A., Mattea C., Stapf S., Ardelean I. (2020). The Effect of Silica Fume and Organosilane Addition on the Porosity of Cement Paste. Molecules.

[24] Pop A., Badea C., Ardelean I. (2013). The Effects of Different Superplasticizers and Water-to-Cement Ratios on the Hydration of Gray Cement Using T2-NMR. Appl. Magn. Reson..

[25] Muller A.C.A., Scrivener K.L., Skibsted J., Gajewicz A.M., McDonald P.J. (2015). Influence of Silica Fume on the Microstructure of Cement Pastes: New Insights from 1H NMR Relaxometry. Cem. Concr. Res..

[26] Meiboom S., Gill D. (1958). Modified Spin-Echo Method for Measuring Nuclear Relaxation Times. Rev. Sci. Instrum..

[27] Kimmich R., Anoardo E. (2004). Field-Cycling NMR Relaxometry. Prog. Nucl. Magn. Reson. Spectrosc..

[28] Rahul A.V., Santhanam M., Meena H., Ghani Z. (2019). 3D Printable Concrete: Mixture Design and Test Methods. Cem. Concr. Compos..

[29] Venkataramanan L., Song Y.Q., Hurlimann M.D. (2002). Solving Fredholm Integrals of the First Kind with Tensor Product Structure in 2 and 2.5 Dimensions. IEEE Trans. Signal Process..

[30] Bede A., Scurtu A., Ardelean I. (2016). NMR Relaxation of Molecules Confined inside the Cement Paste Pores under Partially Saturated Conditions. Cem. Concr. Res..

[31] Faux D.A., McDonald P.J. (2018). A Model for the Interpretation of Nuclear Magnetic Resonance Spin-Lattice Dispersion Measurements on Mortar, Plaster Paste, Synthetic Clay and Oil-Bearing Shale. Microporous Mesoporous Mater..

[32] Faux D.A., McDonald P.J., Howlett N.C. (2017). Nuclear-Magnetic-Resonance Relaxation Due to the Translational Diffusion of Fluid Confined to Quasi-Two-Dimensional Pores. Phys. Rev. E.

[33] Faux D.A., McDonald P.J. (2017). Explicit Calculation of Nuclear-Magnetic-Resonance Relaxation Rates in Small Pores to Elucidate Molecular-Scale Fluid Dynamics. Phys. Rev. E.

[34] Faux D., Kogon R., Bortolotti V., McDonald P. (2019). Advances in the Interpretation of Frequency-Dependent Nuclear Magnetic Resonance Measurements from Porous Material. Molecules.

[35] Faux D.A., McDonald P.J. (2018). Nuclear-Magnetic-Resonance Relaxation Rates for Fluid Confined to Closed, Channel, or Planar Pores. Phys. Rev. E.

[36] Kogon R., Faux D. (2022). 3TM: Software for the 3-Tau Model. SoftwareX.

[37] Kogon R., Faux D. (2021). 3TM: Fitting Software for Fast Field Cycling NMR Dispersion Data. https://zenodo.org/records/5774107.

[38] Pop A., Ardelean I. (2015). Monitoring the Size Evolution of Capillary Pores in Cement Paste during the Early Hydration via Diffusion in Internal Gradients. Cem. Concr. Res..

[39] Roussel N. (2018). Rheological Requirements for Printable Concretes. Cem. Concr. Res..

[40] Michel L., Reiter L., Sanner A., Flatt R.J., Kammer D.S. (2024). Structural Build-up at Rest in the Induction and Acceleration Periods of Portland Cement. Cem. Concr. Res..

[41] Jakob C., Jansen D., Ukrainczyk N., Koenders E., Pott U., Stephan D., Neubauer J. (2019). Relating Ettringite Formation and Rheological Changes during the Initial Cement Hydration: A Comparative Study Applying XRD Analysis, Rheological Measurements and Modeling. Materials.

[42] Scrivener K., Ouzia A., Juilland P., Kunhi Mohamed A. (2019). Advances in Understanding Cement Hydration Mechanisms. Cem. Concr. Res..

[43] Wang B., Faure P., Thiéry M., Baroghel-Bouny V. (2013). 1H NMR Relaxometry as an Indicator of Setting and Water Depletion during Cement Hydration. Cem. Concr. Res..

[44] Jansen D., Neubauer J., Goetz-Neunhoeffer F., Haerzschel R., Hergeth W.-D. (2012). Change in Reaction Kinetics of a Portland Cement Caused by a Superplasticizer—Calculation of Heat Flow Curves from XRD Data. Cem. Concr. Res..

[45] Yuan Q., Zhou D., Huang H., Peng J., Yao H. (2020). Structural Build-up, Hydration and Strength Development of Cement-Based Materials with Accelerators. Constr. Build. Mater..

[46] Dorn T., Hirsch T., Stephan D. (2023). Working Mechanism of Calcium Nitrate as an Accelerator for Portland Cement Hydration. J. Am. Ceram. Soc..

[47] Jiao D., De Schryver R., Shi C., De Schutter G. (2021). Thixotropic Structural Build-up of Cement-Based Materials: A State-of-the-Art Review. Cem. Concr. Compos..

