# Supplementary material for: Relations Between the Printability Descriptors of Mortar and NMR Relaxometry Data
Source: Materials (Basel). 2025 Jun 27;18(13):3070. doi: 10.3390/ma18133070 (PMC12251319; doi:10.3390/ma18133070)
Supplement: Supplementary file 1 [file materials-18-03070-s001.zip › materials-3674078-supplementary.pdf]

materials

MDPI

Supplementary Materials

Relations Between the Printability Descriptors of Mortar and NMR Relaxometry Data

Mihai M. Rusu [1,2] and Ioan Ardelean [1,2,*]

[1] Department of Physics and Chemistry, Technical University of Cluj-Napoca, 400114 Cluj-Napoca, Romania; mihai.rusu@phys.utcluj.ro
[2] EUT+ Institute of Nanomaterials & Nanotechnologies EUTINN, European University of Technology, European Union
* Correspondence: ioan.ardelean@phys.utcluj.ro

Repeatability of CPMG Measurements

During preparation and in E3DPC contexts, from mixing, pumping until extrusion, the material is subjected to various changes such as mechanical stress due to vibrations or shearing, temperature changes, phase redistribution (i.e. lubrication layer formation) and water migration due to bleeding. To emphasize the sensitivity of NMR signal to such effects and to assess the overall repeatability of CPMG measurements, several tests were performed. Since SR1A1 composition demonstrated an optimal balance between extended printability windows and short print onset times, only SR1A1-type mortars were selected for further investigations.

Temperature effects and uncertainty during NMR CPMG measurements

The effect of environment temperature was investigated by maintaining the temperature around the inserted sample at $t_1 = 22\ ^\circ C$ and $t_2 = 35\ ^\circ C$ while repetitively measuring CPMG decays. A repetition delay interval of only 1 minute was selected for increasing the number of measurements under the same conditions for improved statistics. The rest of the measuring parameters (echo time, recycle delay, parameters in the inverse Laplace processing, etc.) were maintained as in the experimental section.

One can follow the temperature effects over T_{2max} in **Figure S1a**. The initial T_{2max} value, measured at ~ 8 min of hydration is the same in both experiments, since the mortar constituents start from the same initial temperatures. However, the rate of decrease in T_{2max} is obviously higher at $t_2 = 35\ ^\circ C$ due to accelerated hydration. To assess the uncertainty in determining T_{2max}, a trendline obtained by a Saviztky-Golay filter was subtracted from the original signal and the resulting deviations were statistically analyzed. The standard deviation was $\sigma\left(\Delta T_{mas}\right) = 0.05\ ms$, while the maximum deviation obtained in the transverse relaxation time was $\Delta T_{2max} = 0.2\ ms$. The similar procedures were performed on the integrated intensity of the capillary peak (**Figure S1b**) which indicates a standard deviation of $\sigma\left(\Delta A\right) = 0.01 \cdot 10^{-2}$ and a maximum deviation $\Delta A = 0.05 \cdot 10^{-2}$.

Repeatability and spatial dependency of the NMR signal in different volumes of hydrating mortars

To investigate how drying effects or moisture distribution across different depths of the hydrating mortar influence the CPMG measurements, 4 different sampling volumes of the same freshly prepared SR1A1 mix were investigated between 20 min to 200 min of hydration under exactly the same experimental conditions. As illustrated in the inset from **Figure S1c,** the height of the sample column and the depth from which CPMG decays were measured (measuring zone is represented with yellow) varied between 45 to 10 mm and 35 to 5 mm respectively. When inspecting the evolution of T_{2max} (**Figure S1c**) and intensity values (**Figure S1d**), one can observe the same dependence on the hydration time. Further on, the transverse time and integrated area are shown to be in the same range when similar sample volumes are measured (Sample 2 vs Sample 3). The transverse time decreases at more shallow depths. We ascribe this to a certain degree of inhomogeneity in the distribution of finer aggregates, trapped voids and cavities. Due to the same reason and to bleeding effects, the intensity may also decrease at higher depths (Sample1). One can observe however that, when the CPMG measuring depth is too shallow (Sample 4), the intensity will also decrease considerably as an artifact due to the improper sample positioning in the

measuring region which leads to lower analyzed volumes. All together suggest the CPMG technique offers a good repeatability and precision in characterizing mortar samples, especially when operating at similar mortar column length and measuring depth. Thus, to minimize surface drying effects, during the experiments where accelerator dosage is changed, the SR1A0, SR1A1 and SR1A3 samples were poured under similar conditions as Sample 2 and Sample 3.

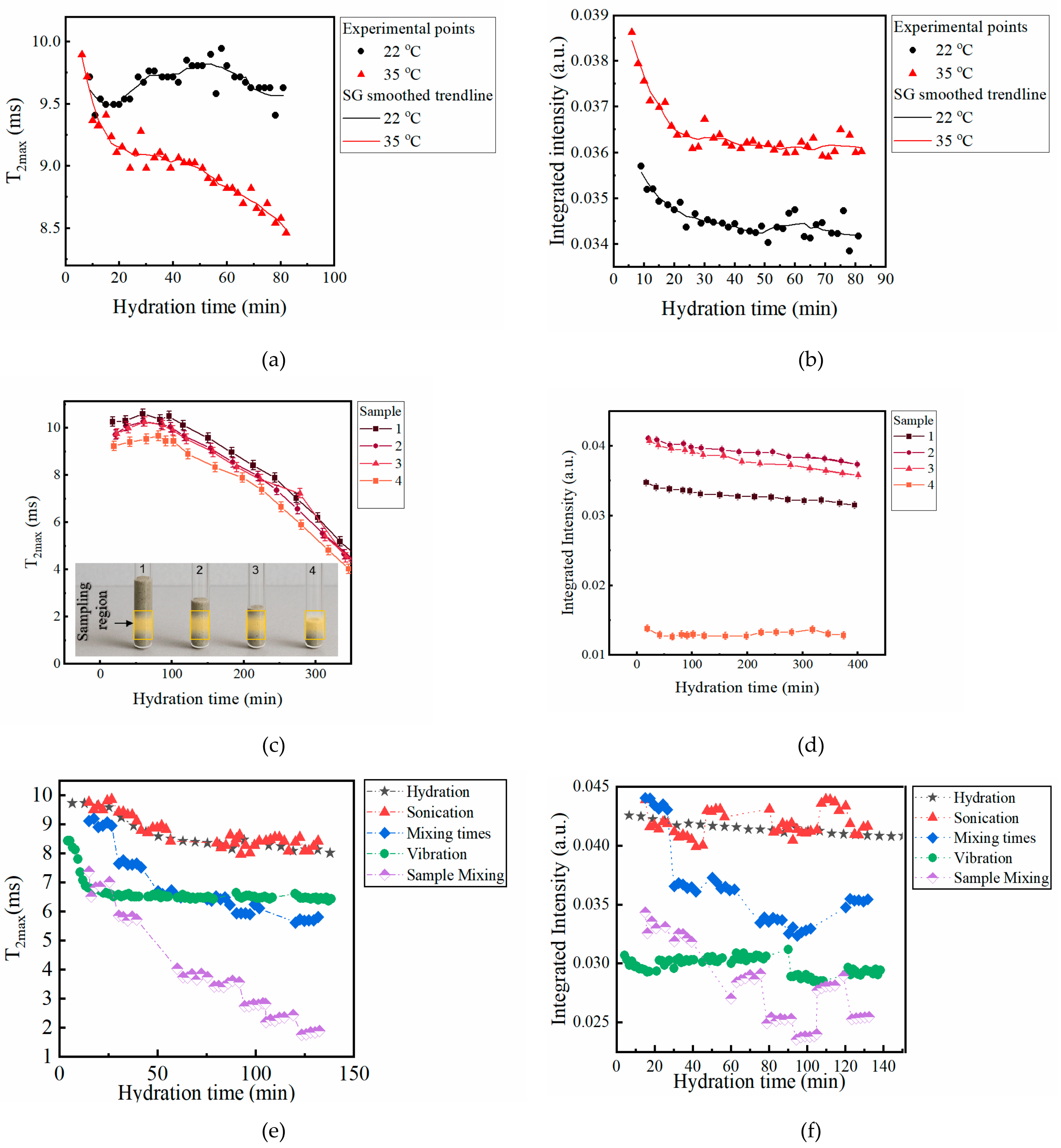

Figure S1. Effects induced over T_{2max} and integrated intensity by temperature (a, b), sampling depth (c, d) and mechanical transformations (e, f) illustrated for SR1A1 mortar

Effects induced by mechanical processing of mortars

The interferences induced by different mechanical transformations of the mortar pastes over T_{2max} and integrated intensity is represented in **Figure S1ef**. The signal obtained during the unhindered hydration of SR1A1

mortar (represented by grey star symbols) is compared with signals obtained after different mechanical interventions were applied periodically (at 5, 20, 30 min until 120 min) during hydration. The experiment where the mortar filled tubes are introduced in ultrasound baths induce small detectable changes (**Figure S1ef blue**) perhaps due to temperature changes, local cavitation and fast restructuration. Changes are more visible in the case where mortar is sampled from the mixing vessel at the given times (**Figure S1ef blue**). The faster decrease in T_{2max} and integrated area is explained by the local temperature increase and losses in paste humidity inflicted by a prolonged exposure to high shear rates. Similar trends are observed when mixing is performed inside the NMR tube (**Figure S1ef purple**). Finally, vibrating the sample decreases the size and number of pores and air bubbles and may add adverse effects if vibration effects is prolonged due to bleeding and segregation. This densification effect of the mortar is well observed in T_{2max} and the integrated intensity values. During the investigations on the effects induced by the accelerator dosage, small mechanical interferences were made in order for the samples to optimally fill the volumes of the NMR tubes, but no vibrations or other similar effects were performed.

UPV tests and temperature readings

The ultrasound pulse velocity (UPV) tests were performed using a MATEST C372M Ultrasonic velocity tester (400MHz) in a two-transducer setup having the maximum resonant frequency of 55kHz. The measurements on fresh mortar samples were performed with a gain setting of 50 dB, voltage amplitude of 200 mV and a sampling duration of 400 ms. The tests performed on hardened mortars were conducted on a set of 5 samples for each investigated composition (using settings of 10 dB). Each sample was measured several times for improved statistics. The temperature readings were recorded from the core of mortar samples. The surface of each sample was sealed with Kapton polyimide films and further introduced in polystyrene to ensure moisture conservation and thermal insulation.

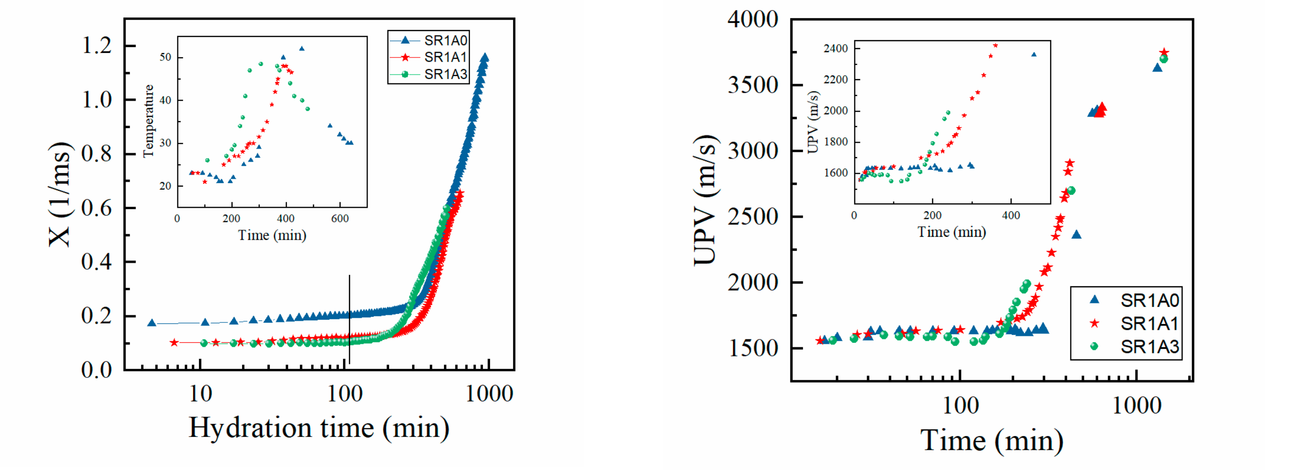

(a) (b)

Figure S2. Paralell between the evolution of relaxation rate (a) and ultrasound pulse velocity measured during UPV tests. The inset plots from (a) and (b) represent the temperature measurements during dormancy, acceleration and deceleration stages and UPV values recorded at smaller time scales.

The exponential-like increase in relaxation rate corresponds with the time at which the temperature of the mortar starts to increase (See inset in **Figure S2**). As described earlier, the relaxation rate is correlated with the surface to volume ratio of capillary pores. It can thus be linked with the structuration rate and the increase in mechanical properties such as elastic modulus and compressive strength and can provide insight into the evolution stages in cement hydration. A first insight into the mechanical properties can be also obtained from UPV tests.

As observed in **Figure S2b,** the trends induced by the mixed use of superplasticizer and accelerator under different amounts that are observed for relaxation rates are consistent with the ones observed when analyzing UPV values. One should also note that the samples exhibited similar UPV values close to 3600 m/s at 1d which reflects a good quality of the investigated compositions.
